# Evaluation of the Antitumor Activity of Quaternary Ammonium Surfactants

**DOI:** 10.3390/ijms242417237

**Published:** 2023-12-07

**Authors:** Kinga Hyla, Dominika Jama, Aleksandra Grzywacz, Tomasz Janek

**Affiliations:** Department of Biotechnology and Food Microbiology, Wrocław University of Environmental and Life Sciences, 51-630 Wrocław, Poland; kinga.hyla@upwr.edu.pl (K.H.); dominika.jama@upwr.edu.pl (D.J.); 114950@student.upwr.edu.pl (A.G.)

**Keywords:** surfactants, quaternary ammonium surfactants, liposomes, tumors, apoptosis

## Abstract

Quaternary ammonium surfactants, due to their diverse chemical structure and their biological properties, can be used in medicine as DNA carriers, disinfectants, and antimicrobial and antitumor agents. In this study, using melanoma A375, colon adenocarcinoma HT-29 and normal human dermal fibroblast (NHDF) cells, we tested the hypothesis that the quaternary ammonium surfactants 2-dodecanoyloxyethyl)trimethylammonium bromide (DMM-11), 2-dodecanoyloxypropyl)trimethylammonium bromide (DMPM-11) and 2-pentadecanoyloxymethyl)trimethylammonium bromide (DMGM-14) act selectively against cancer cells. The results showed that these compounds led to the initiation of the apoptotic process of programmed cell death, as evidenced by the ratio of the relative expression of Bax protein to Bcl-2. The encapsulation of surfactants in liposomes allowed lower concentrations to be used. Moreover, encapsulation reduced their toxicity towards non-cancerous cells. The anticancer efficiency and apoptotic effect of the liposomal formulations with surfactants (DMM-11, DMPM-11 and DMGM-14) were higher than those of surfactant-free liposomes. Therefore, quaternary ammonium surfactant-loaded liposomes show significant potential as delivery vehicles for the treatment of melanoma and colon cancers. The use of nano-formulations offers the advantage of optimizing quaternary ammonium surfactant delivery for improved anticancer therapy.

## 1. Introduction

Melanoma is a skin cancer that has a high mortality rate and develops from melanocytes that produce melanin [1]. This type of cancer is associated with a variety of risk factors, including UV exposure and genetic conditions. Patients who develop metastases and have a prognosis of about 5 years account for less than 20% [2]. Undetected and untreated at an early stage, it quickly spreads to lymph nodes and nearby organs. Surgical treatment, unfortunately, has no positive effect when metastasis is present. Therefore, there is a great need for new agents and therapeutic strategies [1,2,3]. Colorectal cancer accounts for 10.2% of all cancer cases and 9.2% of cancer deaths [4]. According to the National Cancer Institute (NCI), the number of new cases and deaths from colorectal cancer is expected to reach 51,030 and 52,580, respectively, by the end of 2022. Despite advances in the early diagnosis of colorectal cancer, an effective and comprehensive treatment method has still not been developed. The early stage is removed via surgical treatment [5]. Factors that increase the risk of colorectal cancer include genetics and environmental factors. Special attention should be paid to obesity, alcohol consumption, and processed meat [6].

Most of the cytostatic drugs currently used in anticancer therapies induce cell apoptosis. This is the main pathway of programmed cell death, responsible for cellular homeostasis and tissue development [7]. One of the new approaches to cancer therapy is the use of lipophilic drugs that are “swept up” by liposomes, but by accumulating inside them can cause their permeabilization [8]. Lysosomotropic surfactants are a very interesting group of therapeutic molecules characterized by amphiphilic properties, which are currently used as antibiotics [9]. Lysosomotropic compounds are capable of permeabilizing the membrane of lysosomes. Most of these agents were able to penetrate inside the lysosome and accumulate in it; this can lead to activation of cell death [10]. Quaternary ammonium compounds (QACs) contain a moderately basic amine group, are capable of passive diffusion across biological membranes, and their action is primarily focused on lysosomes [11]. The biological activity of synthetic long-chain QACs is well known [12], including antimicrobial activity [13] and activity in several tumor cell lines [14]. In view of the fact that the toxicity of long-chain QACs towards normal mammalian cells is high, other methods of delivering them are being sought. One possibility is the use of soft analogs [15]. Another promising method to reduce toxicity is the use of nanomedicines in the form of liposomes, which provide a platform for encapsulating hydrophobic, hydrophilic, and amphiphilic compounds [16,17,18,19]. Liposomes are carriers for enhanced bioavailability and sustained release. They reduce the toxicity of drugs, improve their stability, and extend their duration of action. Nowadays, they are used for administration of antibiotics, antifungal agents and cytostatics [20].

This study investigated the effect of quaternary ammonium surfactants, particularly their liposomal forms, on chosen cell lines. The 50% inhibitory concentration (IC_50_) of each compound was identified, and these values were considerably lower than those recorded in a previous study [14]. In addition, the study found that the liposomes of these compounds were less cytotoxic toward normal cell lines than cancer cell lines, opening up optimistic prospects for their future use in therapy. Moreover, the use of quaternary ammonium surfactants is in line with the current tendency of medicines that induce apoptosis, as attested through the use of RT-qPCR analysis. The study presents an innovative approach to curing illnesses by utilizing liposome carriers, which augments the drug’s bioavailability and minimizes its damage to non-cancerous cells. This factor holds significance when devising potential anticancer treatments.

## 2. Results and Discussion

### 2.1. MTT Assay

**Figure 1:**
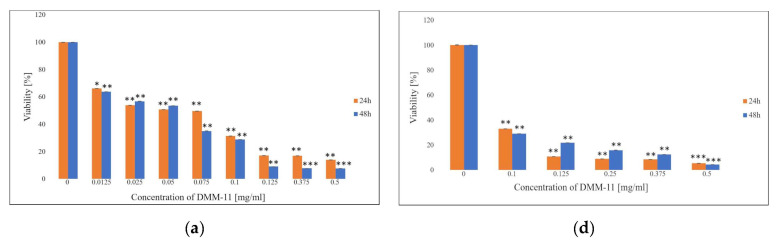

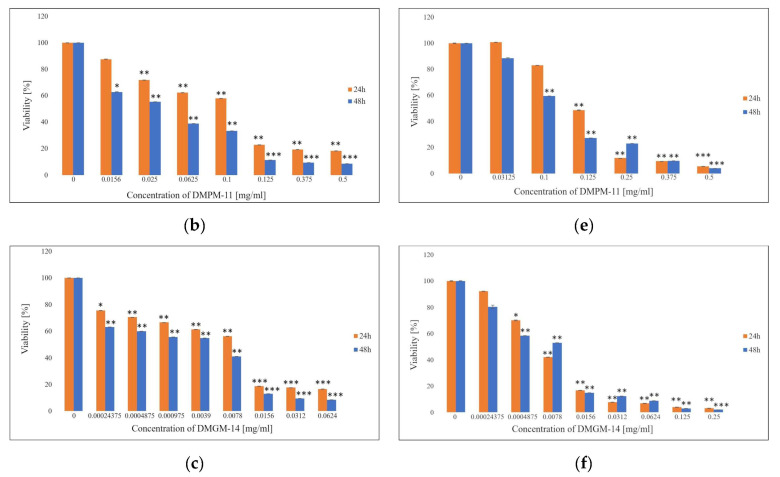
Proliferation rate of A375 melanoma cells as measured via 3-(4,5-dimethylthiazol-2-yl)-2,5-diphenyltetrazolium bromide (MTT) assay. A375 cells (**a**) were treated with 0.0125–0.5 mg/mL concentrations of DMM-11, (**b**) were treated with 0.0125–0.5 mg/mL concentrations of DMPM-11, (**c**) were treated with 2.4 × 10^−4^–0.0624 mg/mL concentrations of DMGM-14, (**d**) were treated with 0.01–0.5 mg/mL concentrations of DMM-11-encapsulated liposomes, (**e**) were treated with 0.03125–0.5 mg/mL concentrations of DMPM-11-encapsulated liposomes, (**f**) were treated with 2.4 × 10^−4^–0.0624 mg/mL concentrations of DMGM-14-encapsulated liposomes for 24 h or 48 h. Cells were incubated with MTT, and the number of viable cells was measured spectrophotometrically at 570 nm. The bars represent the means ± standard deviations (SD) of triplicate values for independent experiments. * 0.05 > *p* > 0.01, ** 0.01 > *p* > 0.001, *** *p* < 0.001.

**Figure 2:**
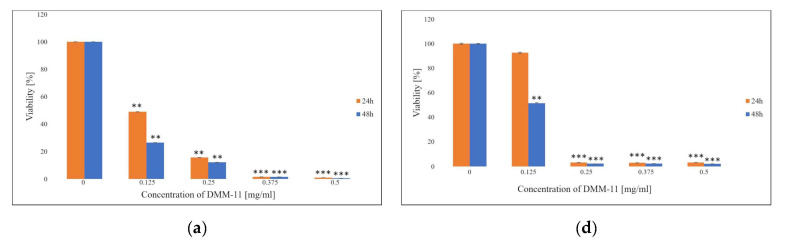

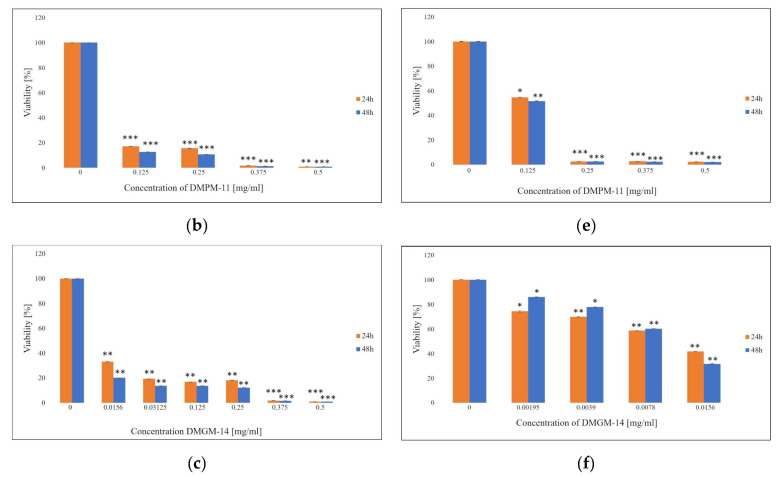
Proliferation rate of HT-29 melanoma cells as measured via MTT assay. HT-29 cells (**a**) were treated with 0.0125–0.5 mg/mL concentrations of DMM-11, (**b**) were treated with 0.0125–0.5 mg/mL concentrations of DMPM-11, (**c**) were treated with 2.4 × 10^−4^–0.0624 mg/mL concentrations of DMGM-14, (**d**) were treated with 0.01–0.5 mg/mL concentrations of DMM-11-encapsulated liposomes, (**e**) were treated with 0.03125–0.5 mg/mL concentrations of DMPM-11-encapsulated liposomes, (**f**) were treated with 2.4 × 10^−4^–0.0624 mg/mL concentrations of DMGM-14-encapsulated liposomes for 24 h or 48 h. Cells were incubated with MTT, and the number of viable cells was measured spectrophotometrically at 570 nm. The bars represent the means ± SD of triplicate values for independent experiments. * 0.05 > *p* > 0.01, ** 0.01 > *p* > 0.001, *** *p* < 0.001.

**Figure 3:**
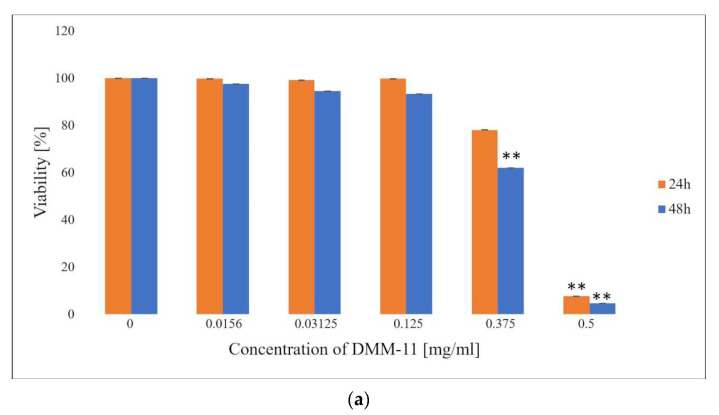

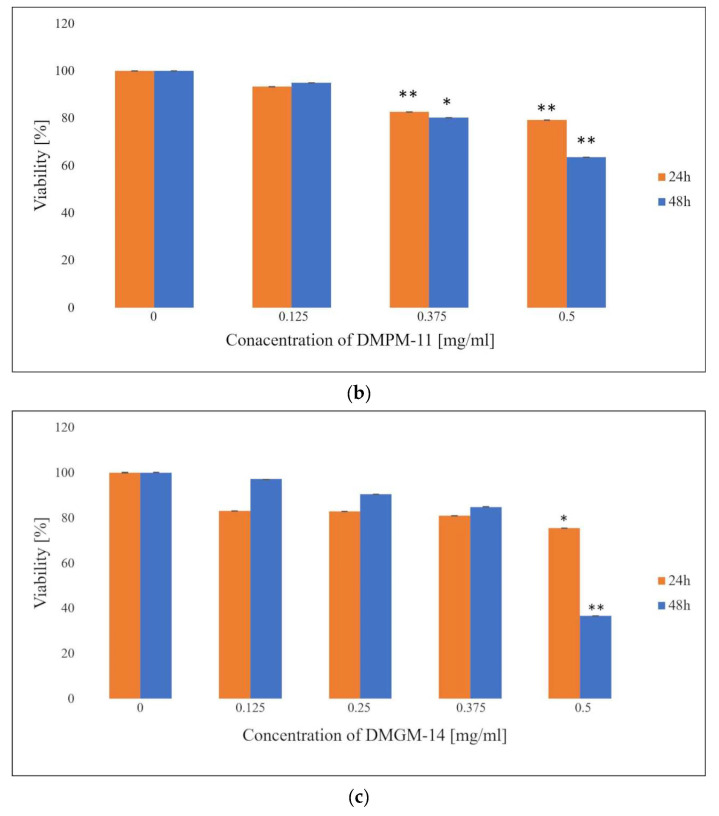
Proliferation rate of normal human dermal fibroblast (NHDF) cells as measured via MTT assay. NHDF cells (**a**) were treated with 0.0156–0.5 mg/mL concentrations of DMM-11-encapsulated liposomes, (**b**) were treated with 0.125–0.5 mg/mL concentrations of DMPM-11-encapsulated liposomes, (**c**) were treated with 0.125–0.5 mg/mL concentrations of DMGM-14-encapsulated liposomes for 24 h and 48 h. Cells were incubated with MTT, and the number of viable cells was measured spectrophotometrically at 570 nm. The bars represent the means ± SD of triplicate values for independent experiments. * 0.05 > *p* > 0.01, ** 0.01 > *p* > 0.001.

In this study, the effects of DMM-11, DMPM-11, and DMGM-14 in the form of free compounds and their liposome forms on the proliferation of A375 melanoma cells (Figure 1), colorectal HT-29 cells (Figure 2) and for the liposome form on NHDFs (Figure 3) were investigated.

Different concentrations were exposed to all cell lines for the indicated times of 24 h and 48 h and their IC_50_ values were determined. Furthermore, MTT data show that treatment with the free compounds, as well their liposome formulations, significantly reduced the survival of A375 and HT-29 cancer cells. IC_50_ doses for A375 were 0.01875 mg/mL (DMM-11), 0.0156 mg/mL (DMPM-11), 0.00195 mg/mL (DMGM-14) for free compounds (Figure 1a–c) and 0.35 mg/mL (DMM-11), 0.425 mg/mL (DMPM-11), 0.4375 mg/mL (DMGM-14) for compounds encapsulated in liposomes (Figure 1d–f). For HT-29, the determined IC_50_ values took the following values for compounds in free form: 0.125 mg/mL (DMM-11), 0.09 mg/mL (DMPM-11), 0.0156 mg/mL (DMGM-14) (Figure 2a–c) and for liposome forms 0.125 mg/mL (DMM-11), 0.125 mg/mL (DMPM-11) and 0.0156 mg/mL (DMGM-14) (Figure 2d–f).

For NHDFs, the obtained IC_50_ values for compounds encapsulated in liposomes were 0.350 mg/mL (DMM-11), 0.6 mg/mL (DMPM-11), and 0.2375 mg/mL (DMGM-14) (Figure 3a–c). The study showed that the liposome forms can deliver a significantly higher dose of the tested compounds compared to the use of their free forms for all cell lines used. In addition, the data collected indicate that the compounds used are considerably less toxic to NHDFs than to A375 and HT-29.

QACs have been reported to significantly induce cell death [21]. QAC-based surfactants have been shown to induce apoptosis in both normal and cancer cells [22]. Chloroquine (CQ) is one of the used lysosomotropic drugs that significantly affect the sensitivity of HT-29 cells, as proven by studies. The authors examined what effect a concentration of 10 μmol/L has on cell survival. In their study, they documented that CQ alone affects the decrease in cell proliferation, as well as helping to sensitize cells to other apoptosis-inducing agents [23]. Likewise, Pan et al. (2019) also confirmed cytotoxic effects on HT-29 cells, with *Ganoderma lucidum* polysaccharide showing a substantial decrease in the viability of cells treated with different concentrations compared to an untreated control. In that study, the IC_50_ value was 10 mg/mL [24]. Previous studies have also shown the effects of 7-hydroxydehydronuciferine (7-HDNF) on cancer cells. Cytotoxicity was tested on the A375 and NHDF models, and a statistically significant effect was observed for A375 cells. NHDFs did not give such a potent response to 7-NDNF, which is considered a satisfactory result [25]. Liposomes are non-toxic and biocompatible, with many advantages, such as the ability to carry hydrophilic and lipophilic agents [20]. Additionally, other synthetic surfactants such as ammonium lauryl sulfate (ALS), sodium lauryl sulfate (SLS), and sodium laureth sulfate (SLES) showed cytotoxic properties in an MTT assay on A375 cells [26]. The use of liposome formulations increases the bioavailability of potential drugs and reduces their toxicity toward normal and cancer cells [27]. Previous research has confirmed that certain surfactants, such as alkyl N,N-dimethyl-alaninates methobromides (DMALM-12) and alkyl N,N-dimethylglycinates methobromides (DMGM-12), possess cytotoxic properties upon exposure to the HT-29 cell line [28]; their IC_50_ values were 79.53 and 3.46 μmol/L, respectively. However, our results were lower than those obtained by Rojewska et al., suggesting that HT-29 cells are highly sensitive to the selected surfactants [28]. Other cationic surfactants tested for cytotoxicity against CHO-K1 cells include cetyltrimethylammonium bromide (CTAB), (16-mercaptohexadecyl)trimethylammonium bromide (MTAB), and N,N,N-trimethyl-3,6,9,12,15-pentaoxaheptadecyl-17-sulfanyl-1-ammonium bromide (POSAB) [29]. Their IC_50_ values were determined to be 14, 30, and >10,000 μM, respectively. All concentrations within the sample significantly exceeded the levels used in our investigation. This indicates that the compounds utilized in our study are more effective and less toxic. In their research, Khowdairy et al. investigated the cytotoxic effects of quaternary ammonium surfactants on different kinds of tumors [30]. They carried out tests on MCF7 (breast carcinoma), HEPG2 (liver carcinoma), U251 (brain tumor), HCT116 (colon carcinoma), and H460 (lung carcinoma) cell lines using dodecylammonium disulfato-cobaltate [30]. The results they obtained ranged from 9.4 to 8.7 μg/mL, which is similar to the concentration of free compounds that we used in our study. It suggests that compounds containing hydrocarbon chains in the range of C-11 to C-14 are capable of being cytotoxic toward cancer cells. All studies conducted on lysosomotropic compounds confirmed the results obtained for DMM-11, DMPM-11, and DMGM-14. They have a significant effect on the survival of A375, HT-29 and NHDF cells. Surfactants in free form have a considerably stronger effect and very low concentrations compared to the literature references. On the other hand, as several authors have pointed out, the encapsulation of the drugs in liposomes can mitigate their effect on the cell due to the gradual release of the medicine. It is hypothesized that the length of the bicarbonate chain plays a crucial role in cytotoxicity. Based on our research, DMGM-14, which contains a C-14 chain, exhibits significantly greater toxicity to cancer cells than surfactants containing a C-11 chain length (DMM-11 and DMPM-11). This applies to both free compounds and those encapsulated in liposomes.

### 2.2. Gene Expression (RT-qPCR)

**Figure 4:**
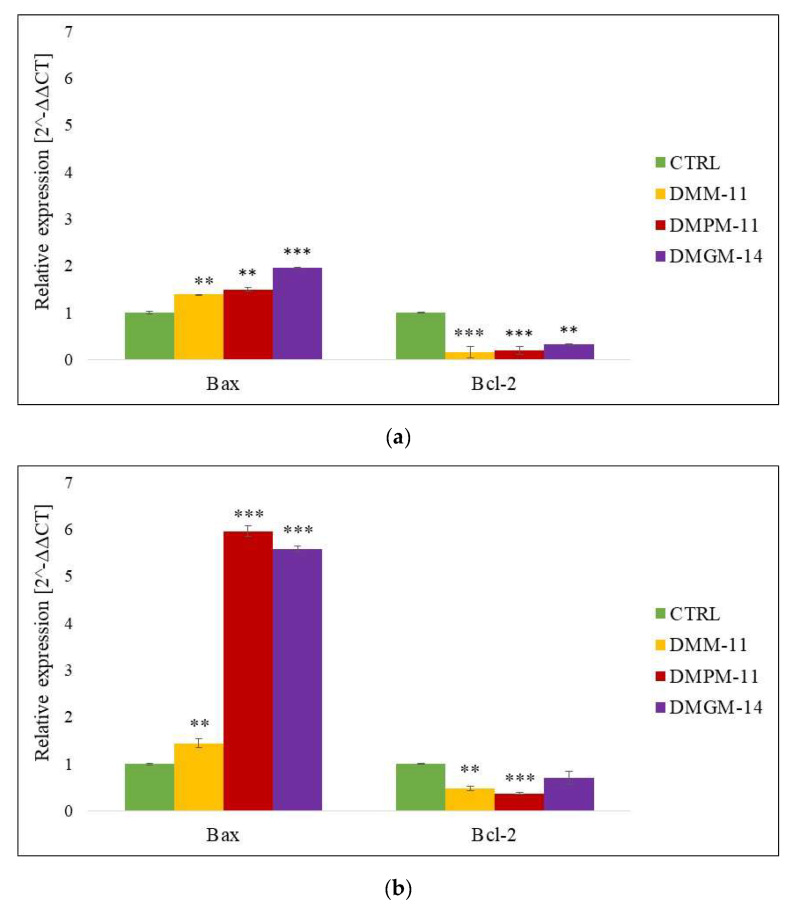
Expression of pro- and anti-apoptotic genes in A375 melanoma cells. A375 cells were treated (**a**) with DMM-11 (0.09375 mg/mL), DMPM-11 (0.0156 mg/mL), DMGM-14 (0.00195 mg/mL) and (**b**) with DMM-11-encapsulated liposomes (0.0875 mg/mL), DMPM-11-encapsulated liposomes (0.1125 mg/mL), DMGM-14-encapsulated liposomes (0.00585 mg/mL) for 24 h. Bar charts illustrating the relative expression of apoptotic markers: *Bax* and *Bcl-2* genes. The bars represent the means ± SD of triplicate values for independent experiments. The significance of differences between treatment groups and untreated cells is shown by an asterisk (*). ** 0.01 > *p* > 0.001, *** *p* < 0.001.

**Figure 5:**
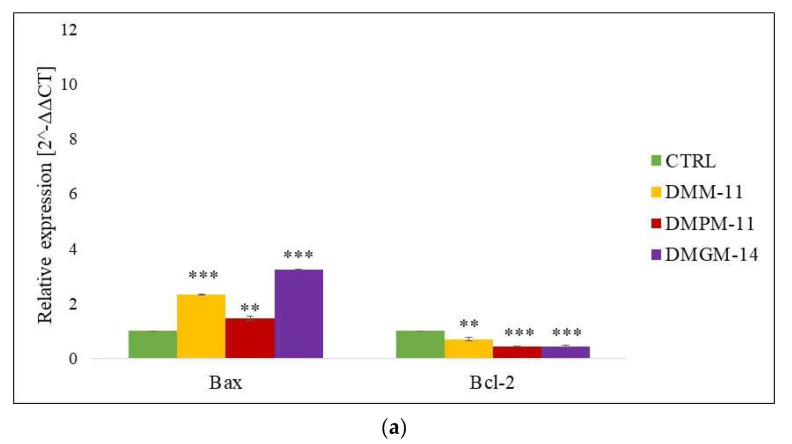

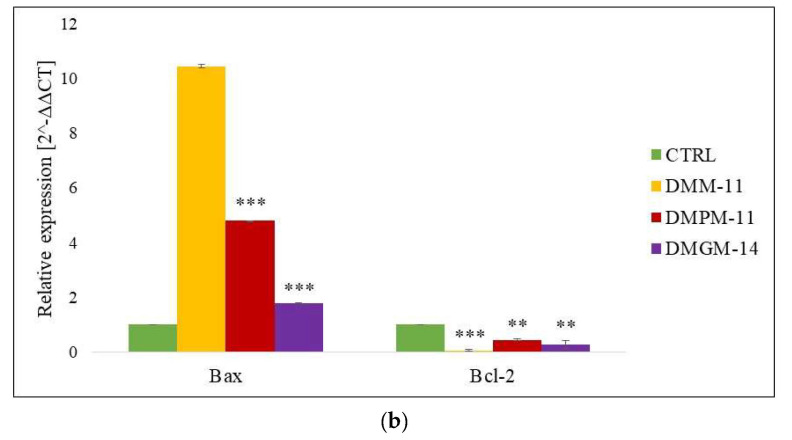
Expression of pro- and anti-apoptotic genes in colorectal adenocarcinoma HT-29 cells. HT-29 cells were treated (**a**) with DMM-11 (0.09375 mg/mL), DMPM-11 (0.0156 mg/mL), DMGM-14 (0.00195 mg/mL) and (**b**) with DMM-11-encapsulated liposomes (0.0875 mg/mL), DMPM-11-encapsulated liposomes (0.1125 mg/mL), DMGM-14-encapsulated liposomes (0.00585 mg/mL) for 24 h. Bar charts illustrating the relative expression of apoptotic markers: *Bax* and *Bcl-2* genes. The bars represent the means ± SD of triplicate values for independent experiments. The significance of differences between treatment groups and untreated cells is shown by an asterisk (*). ** 0.01 > *p* > 0.001, *** *p* < 0.001.

The relative gene expression of pro- and antiapoptotic proteins was assessed using RT-qPCR at the mRNA level. The data obtained show that A375 (Figure 4a), and HT-29 (Figure 5a) cancer cells treated with the test compounds in free form exhibited significant upregulation of one of the key proapoptotic proteins, Bax, compared to untreated cells. In contrast, the apoptosis inhibitor protein Bcl-2 did not show significant expression for either A375 or HT-29. Moreover, in the case of treatment with compounds encapsulated in liposomes for A375 (Figure 4b), much higher expression of the Bax protein was noted for DMPM-11 and DMGM-14, while for HT-29 (Figure 5b), it was noted for DMM-11 and DMPM-11, compared to the free compounds. The results suggest that the used lysosomotropic surfactants showed a strong cytotoxic effect against both tested cancer calls.

Lysosomotropic compounds such as CQ are commonly used as drugs targeting a larger number of cancers [31]. A study from 2014 showed the impact of these compounds on HT-29 cells. They were able to obtain a positive RT-qPCR assay, which indicated the likely onset of apoptosis. There was an increase in Bax protein of about two-fold compared to the control sample and a decrease in Bcl-2 expression [32]. A previous study also showed that thymoquinone (TQ) could induce up- and down-regulation of apoptosis markers such as Bax or Bcl-2. Using TQ as a surfactant led to the overexpression of Bax protein, confirming the ability to induce cell death [33]. Recent studies also reported that 5-fluoroouracil (5-FU), used as a phase-specific anticancer drug, induced apoptotic processes. This was confirmed by the results of the RT-qPCR assay. At a concentration of 5 μM, the authors observed the overexpression of Bax and the down-expression of Bcl-2 for HT-29 cells [34]. Positive apoptosis induction using FBK was observed for A375 cells. At a concentration of 10 μg/mL, Bax gene expression was eight times that of the control [35]. Furthermore, a study from 2022 documented that chloroquine (CQ) acted on A375 melanoma cells, triggering the induction of apoptosis caused by the permeabilization of the lysosome membrane. The onset of apoptosis activation is indicated by the ratio of Bax protein expression relative to the control [36]. The results obtained in previous studies prove that compounds targeting lysosomes are able to induce apoptosis by increasing the expression of the pro-apoptotic gene Bax and reducing the expression of the apoptosis inhibitor Bcl-2. The collated data suggest that DMM-11, DMPM-11, and DMGM-14 hold promise as potential anticancer drugs. All the surfactants we tested show the ability to induce apoptosis.

### 2.3. Migration—Scratch Assay

**Figure 6:**
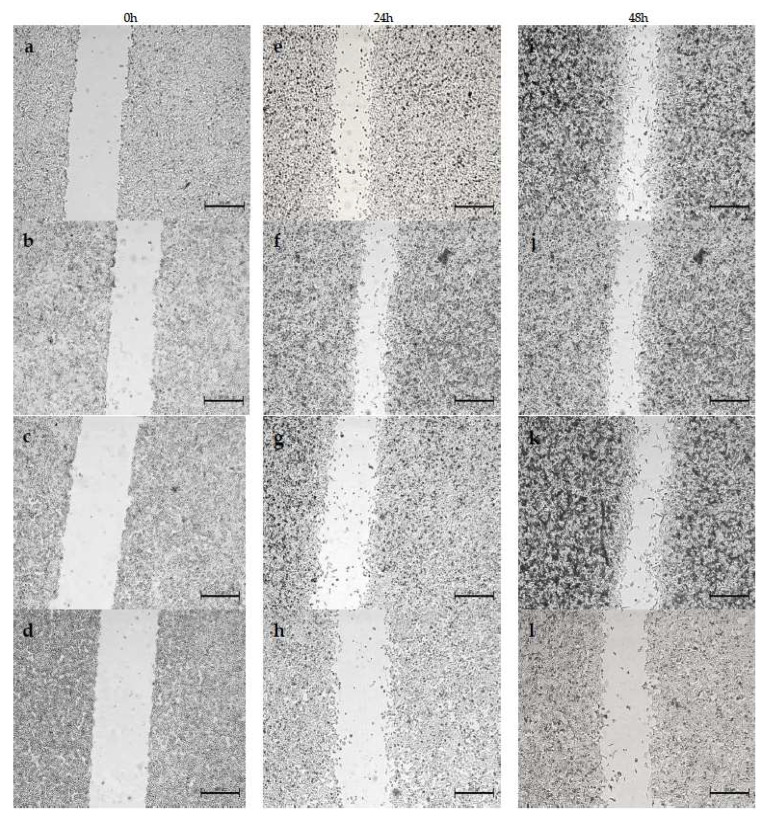
Analysis of A375 cell migration by in vitro scratch assay after 0, 24 and 48 h. A375 cells (**a**,**e**,**i**) constituted a control and were not treated at all, (**b**,**f**,**j**) were treated with DMM-11 at a concentration of 0.01875 mg/mL, (**c**,**g**,**k**) were treated with DMPM-11 at a concentration of 0.0156 mg/mL, and (**d**,**h**,**l**) were treated with DMGM-14 at a concentration of 0.00195 mg/mL. Scale bar—500 µm.

**Figure 7:**
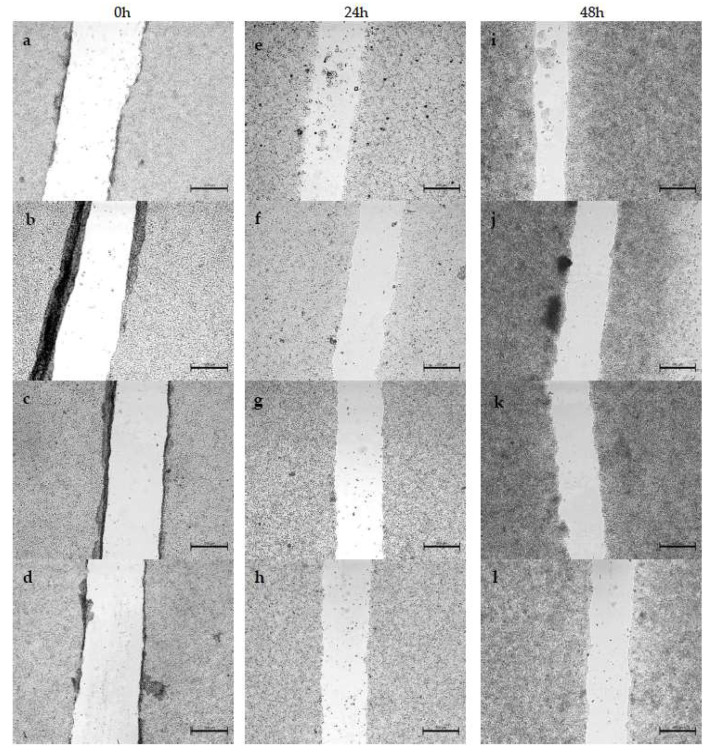
Analysis of HT-29 cell migration by in vitro scratch assay after 0, 24 and 48 h. HT-29 cells (**a**,**e**,**i**) constituted a control and were not treated at all, (**b**,**f**,**j**) were treated with DMM-11 at a concentration of 0.125 mg/mL, (**c**,**g**,**k**) were treated with DMPM-11 at a concentration of 0.09 mg/mL, and (**d**,**h**,**l**) were treated with DMGM-14 at a concentration of 0.0156 mg/mL. Scale bar—500 µm.

**Figure 8:**
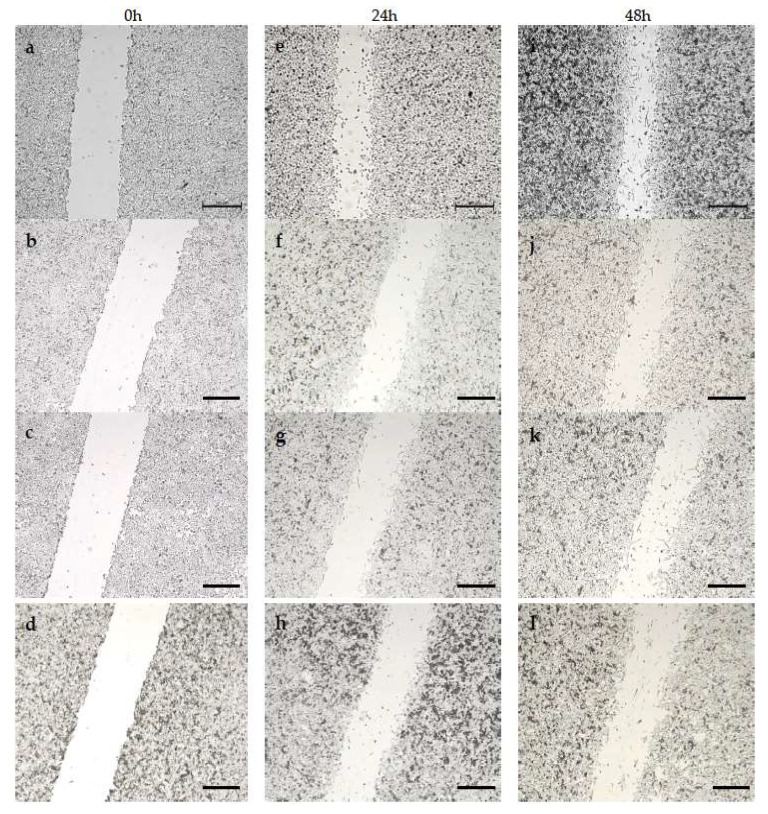
Analysis of A375 cell migration by in vitro scratch assay after 0, 24 and 48 h. A375 cells (**a**,**e**,**i**) constituted a control and were not treated at all, (**b**,**f**,**j**) were treated with DMM-11-encapsulated liposomes at a concentration of 0.0375 mg/mL, (**c**,**g**,**k**) were treated with DMPM-11-encapsulated liposomes at a concentration of 0.025 mg/mL, and (**d**,**h**,**l**) were treated with DMGM-14-encapsulated liposomes at a concentration of 0.001975 mg/mL. Scale bar—500 µm.

**Figure 9:**
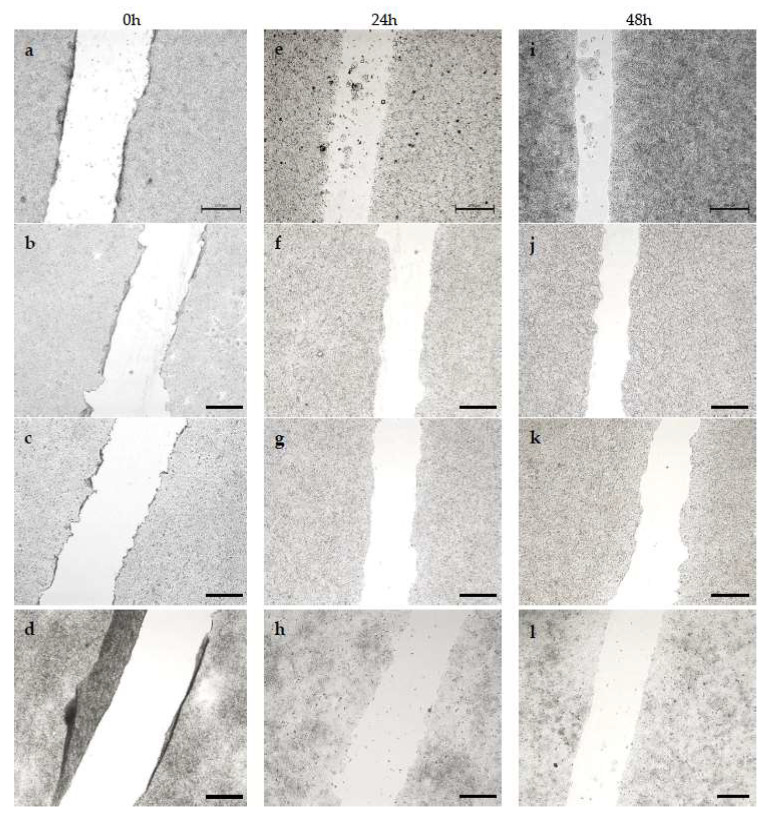
Analysis of HT-29 cell migration by in vitro scratch assay after 0, 24 and 48 h. HT-29 cells (**a**,**e**,**i**) constituted a control and were not treated at all, (**b**,**f**,**j**) were treated with DMM-11-encapsulated liposomes at a concentration of 0.0625 mg/mL, (**c**,**g**,**k**) were treated with DMPM-11-encapsulated liposomes at a concentration of 0.0625 mg/mL, and (**d**,**h**,**l**) were treated with DMGM-14-encapsulated liposomes at a concentration of 0.0197 mg/mL. Scale bar—500 µm.

We evaluated the effect of surfactants in free form and in liposome form on the migration of A375 and HT-29 cells. Measurements were made at 0 h, 24 h and 48 h after adding the compounds to the medium. Control cells migrated noticeably faster than cells treated with the tested compounds, with artificial wound overgrowth for both cell lines used. In addition, a change in morphology was discernible for both A375 and HT-29 treated with surfactants. This may indicate that proliferation is the main factor of invasion in these populations. The data obtained during the study are presented in the form of images, separately for each variant: compounds in free form for A375 (Figure 6) and HT-29 (Figure 7), and the compound encapsulated in liposomes for the A375 melanoma line (Figure 8) and colon cancer line (Figure 9).

Cell migration was significantly more inhibited in HT-29 cells compared to A375 cells, indicating the higher virulence of melanoma. However, both outcomes are acceptable, considering that the migration inhibition was ≥50% over 48 h in specimens treated with various surfactants. By contrast, cell growth in the control sample was around 70% over 48 h of testing. It is hypothesized that the hydrocarbon chain’s length is substantial in determining the compound’s toxicity. Migration inhibition was markedly higher for surfactants with a longer C-14 chain than those with C-11. Furthermore, utilizing liposome formulations augmented the compounds’ efficacy, as demonstrated by the greater number of cells in the wound lumen. Liposomes potentially facilitated better drug assimilation, allowing for gradual release.

Likewise, Khodavirdipour et al. tested a *Syzygium cumini* ethanolic extract (SCE) as a migration inhibitor. The results showed that some compounds were able to inhibit the proliferation of HT-29 [37]. Synthetic surfactants can inhibit cell migration. Effective results were achieved by testing mono-2-ethyhexyl phthalate (MEPH) in the presence of A375 cells [38]. Compared to the commonly used chemotherapeutic agent ciprofloxacin, the results obtained from the wound healing assay are similar [39]. However, we can see a definite difference in cell morphology. Previous studies confirm that surfactants effectively inhibit cell migration. Moreover, they are genuinely comparable with chemotherapeutics, which in turn gives hope for their use as potential drugs.

## 3. Materials and Methods

### 3.1. Cell Culture Conditions

Human colorectal adenocarcinoma HT-29 (ATCC HTB-38) and human melanoma A375 (ATCC CRL-1619) cells were cultured in Dulbecco’s Modified Eagle Medium (DMEM, Institute of Immunology and Experimental Therapy (IITD), Poland) supplemented with 1% glutamine (Sigma-Aldrich, St Louis, MO, USA), 1% antibiotics (10 U/mL penicillin and 10 µg/mL streptomycin, Sigma-Aldrich, St Louis, MO, USA) and 10% fetal bovine serum (FBS, Gibco, USA). Human Normal Dermal Fibroblasts (NHDF; ATCC PCS-201-012) were cultured in α-Minimum Essentials Medium (α-MEM, Institute of immunology and Experimental Therapy (IITD), Poland) supplemented with 1% glutamine (Sigma-Aldrich, St Louis, MO, USA), 1% antibiotics (10 U/mL penicillin and 10 µg/mL streptomycin, Sigma-Aldrich, St Louis, MO, USA) and 10% fetal bovine serum (FBS, Gibco, USA). Cells were grown in a humidified incubator (INCO 105, Memmert GmbH, Schwabach, Germany) at 37 °C, 5% CO_2_ and 95% humidity. Cells were routinely passaged using trypsinization (Trypsin pH 7.2 Institute of Immunology and Experimental Therapy (IITD), Poland). For all experiments, HT-29, A375, and NHDF cells were used at 80% confluence following 2–4 passages.

### 3.2. Cell Treatment

Colorectal adenocarcinoma (HT-29) and human melanoma A375 cells were treated for 24 and 48 h with increasing concentrations of (2-dodecanoyloxyethyl)trimethylammonium bromide (DMM-11) [40] (2-dodecanoyloxypropyl)trimethylammonium bromide (DMPM-11) [40] and (2-pentadecanoyloxymethyl)trimethylammonium bromide (DMGM-14) [41] added to cell culture medium at the following concentrations: DMM-11 was in the range 0–0.5 mg/mL for A375 and HT-29. For DMPM-11, the concentrations were 0–0.5 mg/mL for A375 and HT-29, while DMGM-14 concentrations for A375 were 0–0.0624 mg/mL, but for HT-29 DMGM-14 concentrations were 0–0.5 mg/mL. DMM-11, DMPM-11, and DMGM-14 were dissolved in DMSO to obtain a concentration of 50 mg/mL. Tissue cultures were also treated with surfactants encapsulated in liposomes. The concentration of DMM-11 was 0–0.5 mg/mL for A375, HT-29 and NHDFs, of DMPM-11 was 0–0.5 mg/mL for A375, HT-29 and NHDFs, and DMGM-14 concentrations were 0–0.25 mg/mL for A375, 0–0.0156 mg/mL for HT-29, and 0–0.5 mg/mL for NHDFs. Surfactants encapsulated in liposomes were dissolved in water to obtain a concentration of 1 mg/mL. The structures of DMM-11, DMPM-11 and DMGM-14 are shown in Supplementary Figure S1.

### 3.3. Preparation of Liposomes

Surfactant-loaded liposomes were prepared via the ethanol injection method. The ratio of phosphatidylcholine (PC, Avanti, Polar Lipids, Inc., Alabaster, AL, USA) and surfactants was 3:1. In this experiment, 90 mg of PC was dissolved in 15 mL of 96% ethanol (Sigma-Aldrich, Saint Louis, MO, USA). The ethanol solution was injected into 10 mL of a water solution of surfactants under constant stirring at 200 rpm at an injection rate of 1 mL/min. The ethanol was removed using a vacuum evaporator (Hei-VAP Advantage, Heidolph, Germany) under vacuum at 220 mbar and 70 °C for 20 min with stirring at 210 rpm. The final concentration of PC and surfactants was 1 mg/mL for all. All formulations were stored in the cold at 4 °C. The liposomes were characterized by measuring their size and polydispersity using Zetasizer Pro (Malvern Panalytical, UK). All measurements were performed three times. The liposomal sizes are shown in Table 1, while the polydispersity indexes (PDI) are shown in Table 2.

### 3.4. MTT Assay

HT-29, A375 and NHDF cells were placed on a 96-well plate at a density of 1 × 10^4^ cells per well. The cells were incubated for 24 h in DMEM and α-MEM medium, respectively. After overnight incubation, the DMEM and α-MEM medium was removed, and then cells were treated with increasing concentrations of DMM-11, DMPM-11 and DMGM-14 and incubated for 24 and 48 h. The cell proliferation rate was determined with the standard MTT (3-(4,5-simethylthiazol-2,5-diphenyltetrazolium bromide) (Sigma, St. Louis, MO, USA) assay procedure. Measurements were conducted in triplicate using absorption at 570 nm on a multi-well plate reader (TECAN, Spark, Switzerland).

### 3.5. RNA Isolation and Transcript Quantification

The cells were incubated on a 6-well plate at a density of 2 × 10^6^ in DMEM medium for 24 h at 37 °C. After overnight incubation, the DMEM medium was removed and replaced with DMEM medium supplemented with DMM-11 (0.125 mg/mL), DMPM-11 (0.09 mg/mL), and DMGM-14 (0.0156 mg/mL) for HT-29 cells. For melanoma A375, the concentrations were as follows: DMM-11 (0.01875 mg/mL), DMPM-11 (0.0156 mg/mL), and DMGM-14 (0.00195 mg/mL). The cell line was incubated for 24 h at 37 °C. The procedure was repeated for compounds encapsulated in liposomes. For HT-29, the concentration was DMM-11 (0.125 mg/mL), DMPM-11 (0.125 mg/mL) and DMGM-14 (0.0156 mg/mL). The concentrations for A375 were as follows: DMM-11 (0.35 mg/mL), DMPM-11 (0.425 mg/mL), DMGM-14 (0.4375 mg/mL). The cells were scraped from the bottom of the microplate using a rubber, and then they were centrifuged at 9000× *g* for 5 min. The pellet was washed with ice-cold PBS twice. RNA was isolated using a Total RNA Mini Plus kit (A&A Biotechnology, Gdansk, Poland) followed by DNase I (Thermo Scientific, Waltham, MA, USA) treatment according to the producer’s instructions. RNA quantities were measured using a Biochrom WPA Bioweve II spectrophotometer (Biochrom Ltd., Cambridge, UK). The isolated RNAs were stored in a freezer at −80 °C. cDNA synthesis was performed using a Maxima First Strad cDNA Synthesis kit for RT-qPCR (Thermo Fisher Scientific). qRT-PCR analyses were performed using the DyNAmo Flash SYBR Green qPCR Kit (Thermo Fisher Scientific) and the CFX Connect Real-Time PCR Detection System (Bio-Rad, Hercules, CA, USA). The real-time PCR program was as follows: 95 °C for 2 min, then 41 cycles at 95 °C for 15 s, annealing for 30 s at a temperature specified for tested primers, and elongation at 72 °C for 15 s. The qPCR results were replicated in 3 independent experiments, and then the statistics were determined. Relative gene expression was normalized to the reference gene glyceraldehyde 3-phosphate dehydrogenase (GAPDH) for A375 and β-actin for HT-29 using the 2^−ΔΔCT^ method. The primers used are shown in Table 3. All experiments were performed at least three times.

### 3.6. Migration—Scratch Assay

The HT-29 and A375 were seeded onto a 6-well plate at a density of 2 × 10^6^ in DMEM medium for 48 h at 37 °C. After overnight incubation, the bottom of the wells was scratched, the DMEM medium was drained, then the cells were treated with DMEM medium supplemented with DMM-11 (0.125 mg/mL), DMPM-11 (0.09 mg/mL), and DMGM-14 (0.0156 mg/mL) for HT-29. For melanoma A375, the concentrations were as follows: DMM-11 (0.01875 mg/mL), DMPM-11 (0.0156 mg/mL), and DMGM-14 (0.00195 mg/mL). The cells were also treated with surfactants encapsulated in liposomes at the following concentrations: DMM-11 (0.0625 mg/mL), DMPM-11 (0.0625 mg/mL), DMM-14 (0.00197 mg/mL) for HT-29. For A375, the concentrations were as follows: DMM-11 (0.0375 mg/mL), DMPM-11 (0.025 mg/mL) and DMGM-14 (0.0019752 mg/mL). The cells were photographed through an inverted microscope (Leica DMi1) at 0 h, 24 h and 48 h treatment. Image analyses were performed with ImageJ version 1.54 g software.

### 3.7. Statistical Analysis

All results were presented as the mean and standard deviation (SD) from at least three independent experiments. The significance of differences was determined using Student’s *t*-test. Statistically significant results were indicated with an asterisk: *p* < 0.05 (*), *p* < 0.01 (**), and *p* < 0.001 (***).

## 4. Conclusions

The study compared the cytotoxicity of three lysosomotropic surfactants, namely DMM-11, DMPM-11, and DMGM-14, towards human colorectal adenocarcinoma HT-29 and human melanoma A375 and NHDFs. We observed the cytotoxicity of the compounds towards selected tumor lines in both free and liposome-encapsulated forms, while the liposomal forms did not exhibit strong toxic properties towards primary cells. Furthermore, these compounds demonstrated the capacity to inhibit cell migration, potentially impeding metastasis. The study also found that they could trigger apoptotic processes by increasing the expression of the proapoptotic protein Bax and reducing levels of the apoptosis inhibitor Bcl-2. Furthermore, the encapsulated surfactants yielded results twice as high as their unencapsulated counterparts.

The study findings demonstrate that the quaternary ammonium surfactants (DMM-11, DMPM-11 and DMGM-14) exhibit potential for use as anticancer medications. Nevertheless, more thorough trials should be undertaken to ensure the comprehensiveness of the outcomes. Encapsulation in liposomes is a particularly encouraging method, as it has a smaller cytotoxic impact on healthy cells compared to current chemotherapy and radiotherapy modalities.

## Figures and Tables

**Table 1:** Size of liposomes.

	Size [nm]			Average Size [nm]	SD
DMM-11	206.8	196.7	183.2	195.56	11.84
DMPM-11	176.8	165.7	163.2	168.56	7.23
DMGM-14	95.10	94.27	93.63	94.33	0.73

**Table 2:** Polydispersity index values.

	PDI			Average Value	SD
DMM-11	0.2512	0.2244	0.2456	0.2404	0.0115
DMPM-11	0.2127	0.2081	0.2080	0.2096	0.0021
DMGM-14	0.1091	0.07784	0.1057	0.0975	0.0140

**Table 3:** Primer sequences for RT-PCR.

**HT-29**		
β-actin	Forward	5′CTGTCTGGCGGCACCACCAT3′
	Reverse	5′GCAACTAAGTCATAGTCCGC3′
Bax	Forward	5′AAGCTGAGCGAGTGTCTCAAGCGC3′
	Reverse	5′TCCCGCCACAAAGATGGTCACG3′
Bcl-2	Forward	5′ATGGCAGCAGTAAAGCAAGCGC3′
	Reverse	5′TTCTCCTGGTGGCAATGGCG3′
**A375**		
GAPDH	Forward	5′CAAGGTCATCCATGACAACTTTG3′
	Reverse	5′GTCCACCACCCTGTTGCTGTAG3′
Bax	Forward	5′CAGAACTGGACAGTAACATGGAG3′
	Reverse	5′CAGTTTGCTGGCAAAGTAGAAAAG3′
Bcl-2	Forward	5′ATGTGTGTGGAGAGCGTCAA3′
	Reverse	5′GAGACAGCCAGGACAAATCAA3′

## Data Availability

The data presented in this study are available upon request from the corresponding author.

## Supplementary Materials

The following supporting information can be downloaded at: https://www.mdpi.com/article/10.3390/ijms242417237/s1.
