# Supplementary material for: Evaluation of the Antitumor Activity of Quaternary Ammonium Surfactants
Source: Int J Mol Sci. 2023 Dec 7;24(24):17237. doi: 10.3390/ijms242417237 (PMC10743841; doi:10.3390/ijms242417237)
Supplement: Supplementary file 1 [file ijms-24-17237-s001.zip › ijms-2729896-supplementary.pdf]

Supplementary Information

Evaluation of the Antitumor Activity of Quaternary Ammonium Surfactants

Kinga Hyla[1], Dominika Jama[1], Aleksandra Grzywacz[1], Tomasz Janek[1,*]

[1] Department of Biotechnology and Food Microbiology, Wrocław University of Environmental and Life Sciences, 51-630 Wrocław, Poland

---

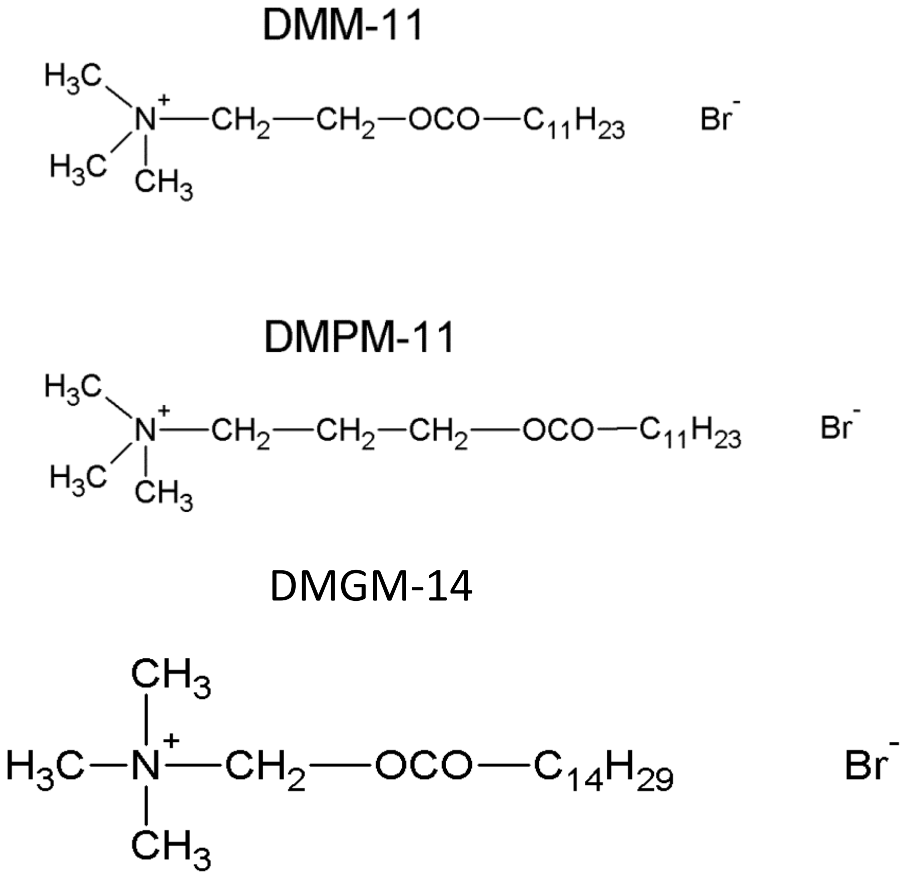

Figure S1. Chemical structures of the surfactants 2-dodecanoyloxyethyl)trimethylammonium bromide (DMM-11), 2-dodecanoyloxypropyl)trimethylammonium bromide (DMPM-11) and 2-pentadecanoyloxymethyl)trimethylammonium bromide (DMGM-14).
